# Hydrochemical, isotopic and microbiota characterization of telese mineral waters (Southern Italy)

**DOI:** 10.1007/s10653-021-00806-4

**Published:** 2021-01-12

**Authors:** Alfonso Corniello, Marco Guida, Luisa Stellato, Marco Trifuoggi, Federica Carraturo, Elena Del Gaudio, Carmela Del Giudice, Giovanni Forte, Antonella Giarra, Marina Iorio, Fabio Marzaioli, Maria Toscanesi

**Affiliations:** 1 Department of Civil, Architectural and Environmental Engineering, Univ. Federico II, P/le Tecchio 80, Napoli, Italy; 2 Department of Biology, Univ. Federico II, Via Cupa Nuova Cintia, 21, Napoli, Italy; 3 Department of Mathematics and Physics, Univ. Vanvitelli, Viale Lincoln, 5, Caserta, Italy; 4 Department of Chemical Sciences, Univ. Federico II, Via Cupa Nuova Cintia, 21, Napoli, Italy; 5 Institute of Marine Sciences (CNR), Calata Porta di Massa, 80, Napoli, Italy

**Keywords:** Telese springs, Italy, Mineral waters, Chemical and isotopic data, Microbiota

## Abstract

The study deals with the analyses of springs and wells at the base of Montepugliano Hill that represents the SE edge of the wide carbonate Matese massif (Campania, southern Italy). At the base of the hill, from west to east and for almost one kilometre, cold springs HCO_3_-Ca type (Grassano springs, ~ 4.5 m^3^/s; TDS: about 0.45 g/L) pass to hypothermal, HCO_3_-Ca type, sulphurous and CO_2_-rich springs (~ 1 m^3^/s with TDS > 1 g/L). Some of the latter are widely used in *Telese Spa* and *Centro Relax Spa. *Chemical and isotopic analyses carried out for this study support the hypothesis that all these waters (mineral and non-mineral) have the same catchment area, which is located in the Matese massif. As regards the sulphurous springs, they receive both meteoric waters infiltration and uprising of deeper waters rich in endogenous CO_2_ and H_2_S gases through important faults systems. Far from these faults, the chemistry of groundwater is scarcely (or not at all) affected by these deep fluid enrichment processes. This scheme is very significant; in fact, when very important groundwater resources are present, it is possible to use both mineral waters in Spa and, in areas far from the faults, those not yet mineralized. Finally, at Montepugliano Hill, in the final stage of the flow path, groundwater is also affected by change in the microbiome: this could provide a basis for comparison between various mineral waters.

## Introduction

In Italy, there are about 480 Spas (distributed in 20 Regions) and the annual budget, also considering their related activities (hotels, restaurants, trade, etc.) exceeds 1.5 billion euros (MEF 2017, 2018). A relevant income of Italian tourism also extensively involves Spas and the cures proposed in these resorts; moreover, their therapeutic efficacy exercises an incisive action for the protection of health in the prevention, treatment and rehabilitation phases.

Campania Region hosts more than 27% of the Italian Spas (MEF 2017, 2018) and most of them (about 120) are located in the volcanic areas W of the city of Naples (Campi Flegrei) and on the Island of Ischia (Distretto Idrografico 2010). Conversely, a dozen of Campania's thermal springs fall at the base of carbonate massifs both inland (Telese, Mondragone, Contursi, etc.) and in coastal areas (Castellammare di Stabia, Scrajo, etc.).

Generally, the assessment of the hydrogeological setting hosting mineral and thermal waters is very complex as it involves the location and definition of the catchment limits (recharge area), its extension, mineralization processes, presence of faults, etc.

For many hydrothermal areas in Campania Region (e.g. Castellammare di Stabia, Scrajo, Mondragone), a good level of knowledge over time has been acquired (Piscopo et al. 1999; Corniello et al. 2013, 2018; Cuoco et al. 2014, 2017; Allocca et al. 2018), which is a prerequisite for a correct and sustainable management of the groundwater resources.

This study deals with the hypothermal, sulphurous springs of Telese (in the Caserta province, Southern Italy), which are located in a carbonate setting and are used in the homonymous *Spa* (one of the most important in Campania) and in the *Centro Relax Spa*. Telese Spa area hosts two springs (Cerro and S. Lucia) and several wells (Diana inf., Diana sup., Goccioloni, S. Stefano). They are widely used for the treatment of skin diseases, problems of the digestive and respiratory systems (Carubbi et al. 2019) and rheumatism. The benefits can be reached by direct assumption (water as a beverage) but also by aerosol and bath therapies. It can be hypothesized that microbial composition is able to enhance the previous described healthy properties, additionally to the peculiar chemical composition.

This study deepens the hydrogeological knowledge of this area, proposing solutions to still open issues (e.g. location of the catchment basin; Corniello and de Riso 1986; Fiorillo et al. 2019), but, above all, it represents a contribution to the knowledge of the groundwater mineralization processes through a multidisciplinary approach that can be also extended to other settings and areas.

In particular, in this work chemical and isotopic analyses of groundwater have been associated with microbiota analysis of waters using *Next Generation Sequencing* approach.

This latest technique, already widely used for groundwater contamination studies (Goldscheider et al. 2006; Pronk et al. 2006, 2009; Bucci et al. 2017; Ender et al. 2018), has found an ever wider application in the study of mineral waters (Weidler et al. 2007; Schulze-Makuch and Kennedy 2008; Aditiawati et al. 2009; Bucci et al. 2011; Tekere et al. 2011; Everroad et al. 2012; Wemheuer et al. 2013; Giovannelli et al. 2016; Hynds et al. 2017; Chaudhuri et al. 2017; Jiang and Takacs-Vesbach 2017; Paduano et al. 2018; Valeriani et al. 2018, 2020) as it is essential to the characterization of each mineral spring providing a basis for comparison with other mineral waters.

## The study area

**Fig. 1:**
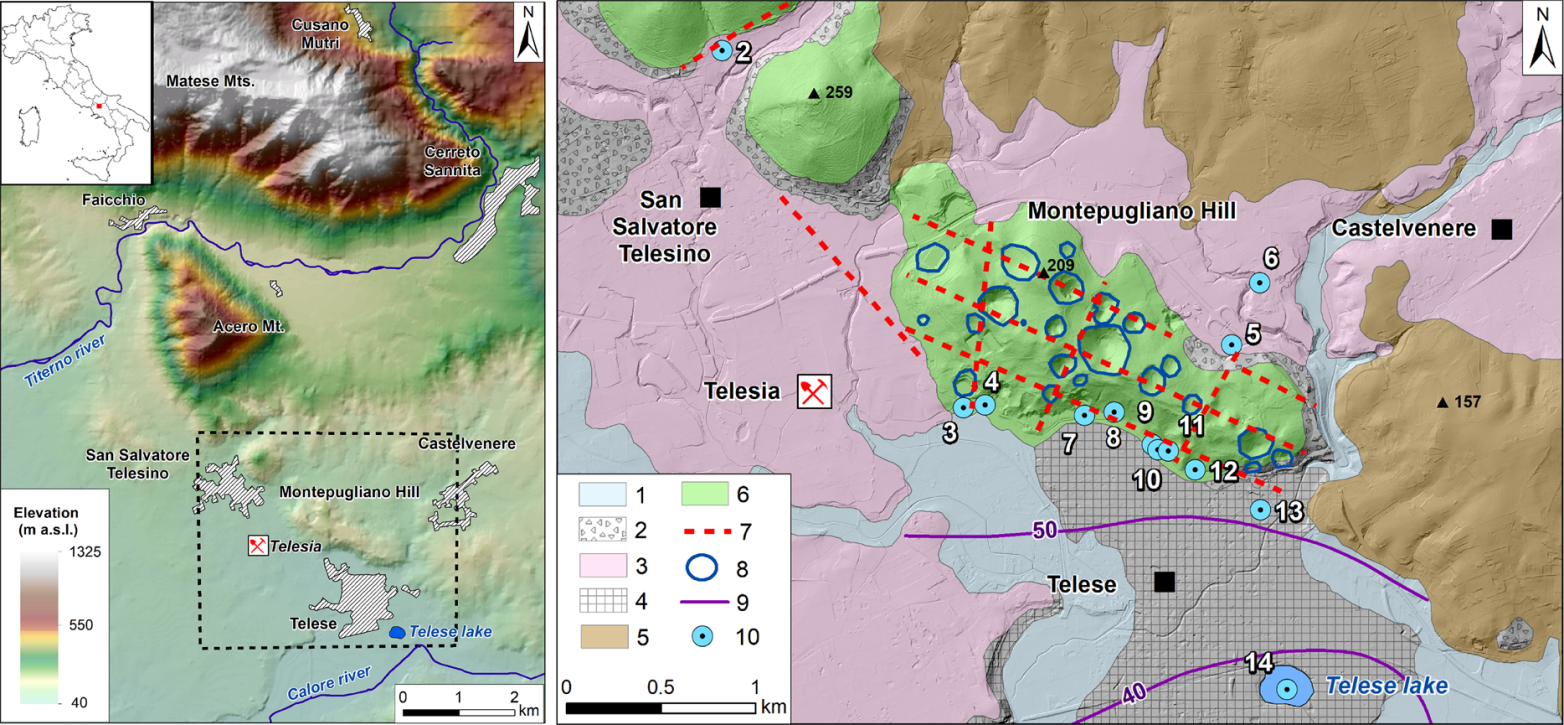
Study area (left) and the hydrogeological map of the Telese area (from Corniello and de Riso 1986 and Fiorillo et al. 2019 mod.). (1) Alluvial deposits (Quaternary); (2) slope deposits; (3) tuff (Campanian Ignimbrite—39,000 y b.p.); (4) travertine; (5) clayey marly flysch; (6) limestones; (7) fault; (8) main sinkholes; (9) groundwater levels—October 2019 (m a.s.l.); (10) sampling points: S. Salvatore well (2); Grassano I spring (3); Grassano II spring (4); Vigne S. well (5); G. Hotel Telese well (6); Centro Relax well (7); Cerro spring (8); S. Lucia spring (9); Diana inf. Well (10); Diana sup. Well (11); Goccioloni well (12); S. Stefano well (13); Telese lake (14)

The study deals with Montepugliano Hill (Campania, southern Italy), which is immediately north of the little town of Telese, and the surrounding areas (Fig. 1).

Montepugliano Hill is made up of Cretaceous limestones, and at its base, very productive springs are present; these outflows are the cold Grassano springs and then, about 800 m eastwards, the hypothermal, sulphurous and CO_2_-rich springs that are widely used in *Telese Spa* and in the *Centro Relax Spa*.

Grassano springs have various outlets, an average discharge of 4.5 m^3^/s and TDS in the order of 0.5 g/L, while the hypothermal springs have discharge of about 1 m^3^/s and TDS > 1 g/L.

Montepugliano Hill is limited to the north and east by low permeable flysch deposits covered by a thick layer of tuff (Campanian Ignimbrite, 39,000 y b.p.; De Vivo et al. 2001); the latter also outcrops extensively to the west of the hill, while it lacks near the springs, where only alluvial deposits and travertines are observed (Fig. 1; Corniello and de Riso 1986).

As shown in Fig. 1, at the top of the hill many sinkholes, some partially covered by tuff, can be identified. The most evident ones (about 20; Fiorillo et al. 2019) have depths between 4 and 98 m. The bottom of the deepest sinkhole is at 78 m a.s.l., while the average value is about 118 m a.s.l. In all the cases, the bottom of the sinkholes is at significantly higher altitudes than those of the various springs. The concentration of sinkholes is higher next to faults (mainly NW–SE oriented) and where these faults are crossed by other orthogonal ones.

To the south of Montepugliano hill, several sinkholes of various sizes are found in travertine; the largest one hosts the Telese lake. Although the ancient roman town of Telesia (cited by Tito Livio in relation to the wars against Hannibal—214 b.C.) is not far from the sulphurous mineral waters, these are not mentioned by the ancient Romans despite their attention to these phenomena. Several authors (Gauthier 1910; Iannichino 1900; Vigliotti 1985) have therefore supposed to link the outflow of mineral springs to the effects of the very violent earthquake occurred in 1349. In particular, the seismic event would have reactivated older mineral springs, as the presence of wide travertine outcrops covered by tuff is certainly linked to the pre-existence of mineral waters.

The relationship between seismic events and mineral waters is also highlighted in more recent times by Harabaglia et al. (2002) who attribute the variations of the SO_4_ ion observed, from 12/1998 to 01/1999, in one of the mineral springs to a seismic swarm.

**Fig. 2:**
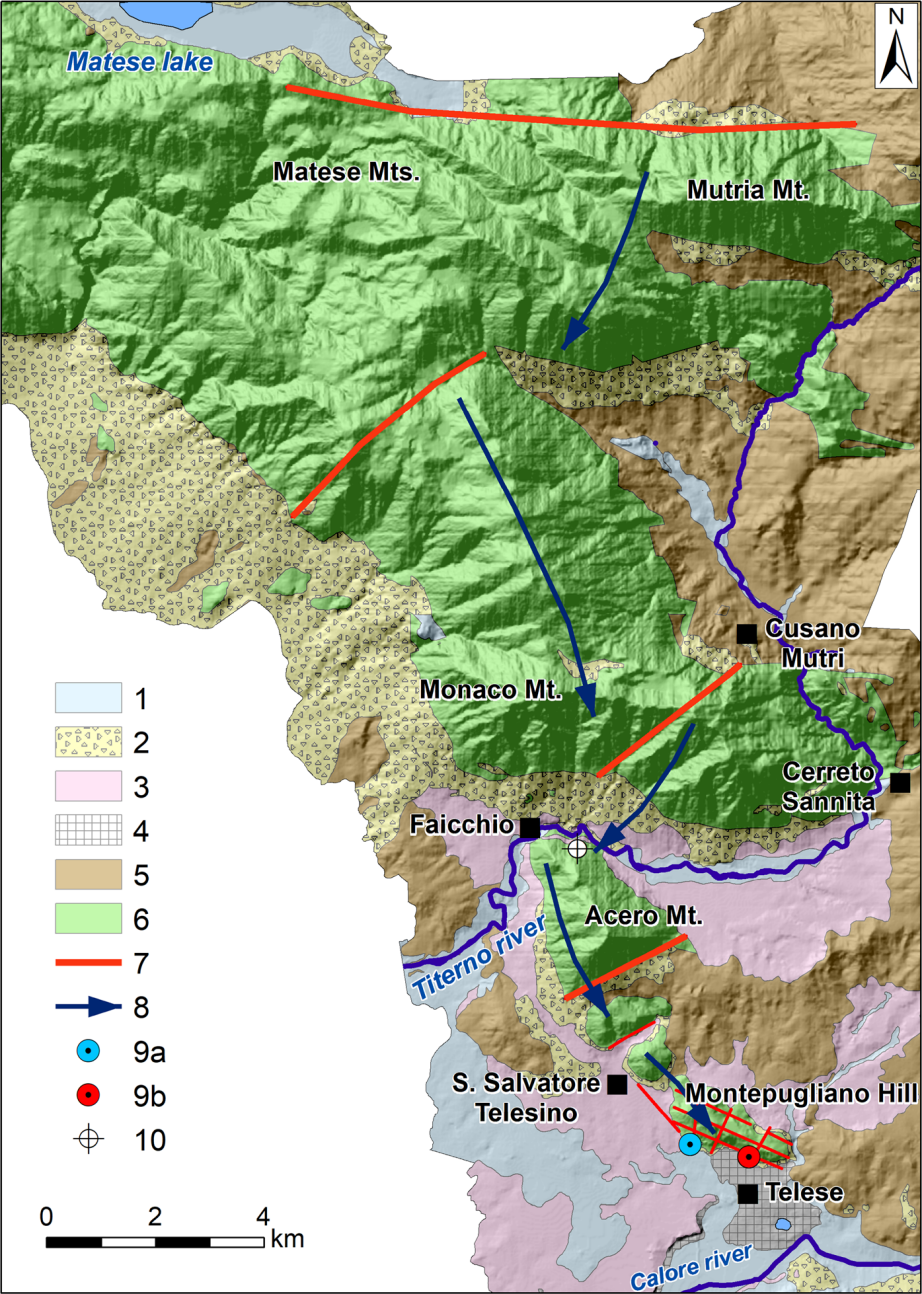
Catchment area of Telese springs (from Corniello and de Riso 1986 mod.). (1) Alluvial deposits; (2) slope deposits; (3) tuff; (4) travertine; (5) clayey marly flysch; (6) carbonates; (7) main fault; (8) groundwater basal flow; (9) Grassano springs **(a)** and mineral wells/springs **(b)**; 10) well 1 (see Table 1)

Montepugliano Hill and the nearest carbonate ridge of Acero Mt. have limited areal extension and this does not justify the abundant discharges of the springs. In fact, previous studies (Corniello and de Riso 1986; Fiorillo et al. 2019) extend the catchment area of the springs to the south-eastern sector of the Matese carbonate massif (Fig. 2). On the other hand, the valley of Titerno River, which morphologically separates Matese massif from Acero Mt. and Montepugliano Hill (Fig. 2), does not hinder the groundwater circulation from Matese, which flows in the carbonate bedrock buried below the alluvial valley, as it is also confirmed by the congruence of the groundwater levels in wells drilled in the bedrock towards NW and SE of Titerno River (Corniello and de Riso 1986).

## Data and methods

The water points coordinates (X–Y–Z) were recorded using a GPS. Groundwater levels were measured in October 2019 in more than 15 wells in the plain from Montepugliano Hill to Calore River to draw the local piezometric surface. Furthermore, temperature, pH and electrical conductivity (EC) were directly measured in situ. During the field campaigns carried out in November 2019, January and February 2020, water samples were collected for chemical, isotopic and bacteriological analyses. The data obtained from analyses were organized in a database in GIS (Table 1) and were also used for drawing thematic maps and graphical elaborations.

**Table 1:** Physico-chemical data of groundwater sampled from springs and wells (location in Figs. 2 and 3); original data for this study (a), from Corniello and de Riso 1986 (b), from ARPAC 2020 (c) and from Harabaglia et al. 2002 (d)

*n*	Date	T °C	pH	Cond	TDS	CO_*2*_	H_*2*_*S*	dH	Na	K	Ca	Mg	Cl	HCO_*3*_	SO_*4*_	NO_*3*_	δ ^18^O	δ D
				µS/cm	mg/L	mg/L	mg/L	°F	meq/L	meq/L	meq/L	meq/L	meq/L	meq/L	meq/L	mg/L	‰	‰
1	01/10/1985 (b)	11.0	7.3	400	236	30	–	23.5	0.3	0.0	3.9	0.8	0.4	4.4	0.2	0.0		
2	10/11/2019 (a)	12.3	7.0	501	346	42.8	< 0.1	27.2	0.4	0.1	4.0	1.5	0.4	4.9	0.2	4.3	−7.3	-40.2
3	01/02/1976 (b)	11.0	6.5	818	510	116	–	49.5	0.3	0.1	8.0	1.9	0.9	9.3	0.1	0.0		
	26/11/2019 (a)	11.7	6.9	800	560	202	< 0.1	41.2	0.6	0.1	6.3	1.9	0.6	8.9	0.2	3.7	−8.5	−47.8
	20/01/2020 (a)	11.6	6.9	800	550	143	5.8	42.8	0.6	0.1	6.6	2.0	0.5	7.2	0.2	5.9		
	20/02/2020 (a)	11.5	6.9	782	570	137	–	57.8	0.6	0.1	6.9	2.1	0.5	8.8	0.2	4.0		
4	20/06/2003 (c)	13.2	–	554	–	–	–	57.0	0.7	0.1	8.8	2.6	0.6	10.5	0.2	1.9		
	02/12/2003 (c)	–	–	794	–	–	–	43.8	0.7	0.1	6.5	2.4	0.6	10.3	0.2	3.3		
	25/05/2005 (c)	13.0	–	991	–	–	–	56.0	0.8	0.1	8.7	2.5	1.1	11.0	0.2	2.0		
	20/09/2005 (c)	12.0	–	877	–	–	–	53.7	0.8	0.1	8.3	2.5	0.8	10.0	0.3	7.0		
	05/06/2007 (c)	14.0	–	1037	–	–	–	49.4	0.7	0.1	7.6	2.3	0.7	9.2	0.2	3.0		
	10/10/2007 (c)	13.0	–	807	–	–	–	50.1	0.7	0.1	7.6	2.4	0.6	9.3	0.2	3.0		
	04/06/2009 (c)	14.0	6.8	894	–	–	–	46.8	0.6	0.1	7.2	2.2	0.5	9.6	0.1	2.7		
	03/12/2009 (c)	10.0	6.8	879	–	–	–	47.8	0.6	0.1	7.4	2.1	0.6	9.3	0.2	3.1		
	16/12/2010 (c)	13.0	7.0	594	–	–	–	48.7	0.6	0.1	7.5	2.3	0.7	9.4	0.2	3.5		
	15/05/2012 (c)	12.0	6.9	802	–	–	–	53.7	0.7	0.1	8.3	2.4	0.5	10.2	0.2	4.6		
	25/10/2012 (c)	12.0	7.0	735	–	–	–	47.7	0.6	0.1	7.4	2.2	0.6	9.4	0.2	3.7		
	26/11/2019 (a)	11.6	6.7	980	700	348	< 0.1	51.1	0.7	0.1	8.1	2.1	0.7	10.9	0.2	2.1	−8.6	−49.3
	20/01/2020 (a)	11.6	6.6	1075	725	254	< 0.1	59.4	0.8	0.1	9.5	2.4	0.8	11.2	0.2	2.1		
	20/02/2020 (a)	11.5	6.6	1036	712	254	–	78.1	0.8	0.1	9.4	2.4	0.8	12.0	0.2	2.8		
5	26/11/2019 (a)	12.5	6.8	1173	820	319	< 0.1	58.1	1.5	0.1	9.3	2.3	1.4	12.8	0.5	1.3	−7.9	−45.7
	22/01/2020 (a)	6.6	6.9	1158	814	183	< 0.1	61.0	1.3	0.1	9.8	2.4	1.2	10.9	0.5	< 1		
	20/02/2020 (a)	8.9	6.9	1150	795	236	–	84.4	1.3	0.1	10.1	2.5	1.2	12.7	0.4	2.0		
6	26/11/2019 (a)	14.4	7.1	969	670	118	< 0.1	40.4	2.4	0.3	6.9	1.2	2.5	7.2	0.9	13.3	−6.5	−37.5
	22/02/2020 (a)	13.6	7.0	1029	705	104	< 0.1	62.1	2.9	0.4	7.0	1.2	3.5	6.7	1.0	17.6		
7	26/11/2019 (a)	15.6	6.5	1862	1303	832	10.0	96.4	2.4	0.2	15.4	3.9	2.3	19.7	0.5	0.9	−8.4	−48.9
	20/01/2020 (a)	14.8	6.4	1780	1293	625	6.4	100.5	2.3	0.2	16.1	4.0	2.2	19.4	0.4	< 1		
	20/02/2020 (a)	12.1	6.3	1750	1282	808	–	130.3	2.2	0.2	15.6	3.8	2.2	20.6	0.4	2.2		
8	01/01/1986 (b)	–	6.3	1340	–	400	–	94.0	0.7	0.1	13.6	5.2	1.6	16.0	0.6	0.0		
	22/01/2020 (a)	13.1	6.5	1217	820	206	< 0.1	66.4	1.2	0.1	10.6	2.6	1.1	11.3	0.5	< 1		
	22/02/2020 (a)	12.9	6.5	1194	815	451	< 0.1	87.5	1.2	0.1	10.5	2.6	1.1	13.2	0.4	3.2		
9	19/11/1998 (d)	20.1	6.1	2330	2424	–	–	–	4.3	0.5	21.2	5.7	3.9	27.2	0.3	0.1		
	26/11/2019 (a)	19.4	6.2	2391	1660	1260	9.8	130.9	4.0	0.4	20.8	5.4	3.8	27.2	0.8	0.9	−8.1	−48.6
	20/01/2020 (a)	19.4	6.1	2505	1710	1077	7.4	136.3	4.3	0.4	21.6	5.7	4.1	25.7	0.7	0.9		
	20/02/2020 (a)	19.4	6.1	2420	1680	1293	6.4	175.3	4.3	0.4	20.8	5.5	4.1	25.9	0.5	< 1		
10	26/11/2019 (a)	19.9	6.2	2338	1636	1572	9.9	129.5	3.9	0.4	20.6	5.3	3.7	25.7	0.8	0.9	−8.2	−48.3
	20/01/2020 (a)	13.4	6.2	2415	1645	1438	6.4	159.0	5.8	0.5	25.0	6.8	5.5	22.4	0.3	< 1		
	20/02/2020 (a)	19.4	6.1	2342	1620	1356	5.9	170.2	4.0	0.4	20.2	5.3	3.8	26.6	0.6	3.1		
11	26/11/2019 (a)	21.9	6.2	2884	2002	1646	12.8	157.8	5.7	0.5	24.9	6.7	5.4	33.5	0.6	0.9	−8.3	−49.6
	20/01/2020 (a)	20.3	6.2	2943	2015	1447	7.7	134.4	4.2	0.4	21.4	5.5	3.9	23.6	0.2	< 1		
	20/02/2020 (a)	21.0	6.2	2793	1980	1686	7.0	204.6	5.6	0.5	24.3	6.6	5.4	28.1	0.3	3.3		
12	26/11/2019 (a)	19.6	6.1	2256	1560	1473	10.4	122.5	3.5	0.3	19.7	4.8	3.3	26.8	0.7	0.9	−8.2	−48.8
	20/01/2020 (a)	19.2	6.2	2316	1520	624	6.7	127.7	3.6	0.4	20.5	5.1	3.4	25.3	0.6	< 1		
	22/02/2020 (a)	19.3	6.2	2213	1509	1114	8.8	168.3	3.6	0.3	20.0	5.0	3.4	24.8	0.5	4.0		
13	01/10/1983 (b)	20.0	6.5	2028	1583	786	–	112.0	5.2	0.5	19.5	4.7	4.5	25.0	0.5	0.0		
	26/11/2019 (a)	19.1	6.3	1730	1220	815	8.1	94.5	2.0	0.2	15.3	3.6	1.9	19.9	0.5	0.9	−8.2	−48.3
	20/01/2020 (a)	18.3	6.2	1801	1240	386	< 0.1	98.6	2.1	0.2	15.9	3.8	2.0	18.6	0.4	< 1		
	20/02/2020 (a)	18.2	6.2	1779	1230	916	6.4	135.8	2.2	0.2	16.2	3.9	2.1	21.5	0.3	3.0		
14	26/11/2019 (a)	16.6	7.9	446	310	57	< 0.1	19.5	0.9	0.1	2.2	1.7	0.8	4.3	0.2	0.9	−0.6	−10.3
	22/01/2020 (a)	8.8	7.7	492	336	127	< 0.1	21.4	0.8	0.1	2.5	1.8	0.8	4.8	0.2	< 1		
	20/02/2020 (a)	10.3	8.0	466	315	74	< 0.1	22.7	0.9	0.1	2.6	1.8	0.8	4.9	0.2	1.2		

1 (Well 1); 2 (San Salvatore well); 3 (Grassano I Spring); 4 (Grassano II Spring); 5 (Vigne Sannite Well); 6 (G. Hotel Telese Well); 7 (Centro Relax Well); 8 (Cerro Spring); 9 (S. Lucia Spring); 10 (Diana inf. Well); 11 (Diana sup. Well); 12 (Goccioloni Well); 13 (S. Stefano Well); 14 (Telese Lake)

Chemical analyses, including major cations (Na^+^, K^+^, Ca^2+^, Mg^2+^), anions (Cl^−^, SO_4_^2−^, NO_3_^−^, HCO_3_^−^) and trace metals (Al, As, B, Ba, Cd, Cr, Cu, Hg, Fe, Li, Mn, Ni, Pb, Sb, Se, Sn, Zn) have been performed by the laboratories of the University of Naples Federico II (Department of Chemical Sciences) by using ion chromatography and mass spectrometry on samples stored at 4 °C. The analyses show a charge balance error of less than 5% (Table 1).

Isotopic analysis (δ^18^O and δD) was performed at the CIRCE (Centre for Isotopic Research on the Cultural and Environmental heritage) laboratory of the Department of Mathematics and Physics of the University of Campania “Luigi Vanvitelli”. Water stable isotopes (δD and δ^18^O, reported as ‰ versus VSMOW) have been measured by means of a gas bench–CF–IRMS system (Delta V Thermo Fisher) and by a TC/EA–CF–IRMS system with an analytical precision of 0.2 ‰ and 1 ‰, for δ^18^O and δD determination, respectively.

Finally, the bacteriological analyses have been carried out by the Hygiene Laboratories of the Department of Biology of the University Federico II of Naples. The extraction of the total DNA from water samples was carried out to sequence the genome of the whole microbiota, employing NGS (next-generation sequencing) technology. Waters samples (2 Liters) were collected and transported to the laboratory in refrigerated conditions (4 °C). The water samples were filtered, and germs concentrated on nitrocellulose membranes (0.22 μm) (Sartorius, USA), under aseptic conditions. The membranes were transferred into sterile 2-mL vials containing 5 g of glass beads; membrane was cut into four parts and CTAB extraction protocol (Doyle 1990) was carried out. The extraction of the total bacterial DNA from the water samples allowed to sequence the genome of all the micro-organisms. The extracted DNA samples were amplified with PCR using the V3 and V4 primers, complementary to V3-V4 variable region of the bacterial 16S rRNA gene (500 bp). Sequencing was performed employing MiSeq Illumina platform, using 2 × 300 bp paired end, 600 cycles, following the manufacturer's instructions (Illumina MiSeq, USA). Differences in the group's communities retrieved from Illumina experiment were assessed by anosim using weighted UniFrac distance and Anova using Bray Curtis distance (Mothur) (Sala-Comorera et al. 2019).

## Results

### Groundwater flow

Recently, Fiorillo et al. (2019) have proposed a hydraulic model, in the MODFLOW environment, of the groundwater flow from the Matese ridge towards Montepugliano Hill; the model shows that:

- Montepugliano constitutes a "drain" for the groundwater flow;
- the value of the hydraulic conductivity (K) of the carbonate aquifer providing the best model calibration is 0.001 m/s;
- groundwater flow is horizontal from Matese massif to Montepugliano Hill and shows an *upwelling phenomenon* at Montepugliano.

It is possible to calculate groundwater flow velocity between Matese and Montepugliano with Eq. 1.

1 $$v = \frac{Ki}{{n_{e} }}$$

where *i* is the hydraulic gradient = 0.0011 (Corniello and de Riso 1986; Fiorillo et al. 2019) and *n*_*e*_ (specific yield) = 0.02 (Walton 1970; Castany 1982; Weigh and Satteregger 2001). Therefore, using the above values the velocity resulted of 4.8 m/day.

At the Montepugliano Hill, velocity is certainly higher due to the karst of the rocks and the presence of many faults; in this case, it may be likely a value even double compared to that indicated.

At the base of the Montepugliano Hill, the Grassano springs have (as already mentioned) the highest discharge: 4.5 m^3^/s, with the minimum value generally recorded in November (Fiorillo et al. 2019). This discharge higher than mineral waters is probably due: *(a)* to the presence of faults that act as drains for groundwater (Fig. 1) and *(b)* to the local lower altitude (about 54 m a.s.l.) of the not very permeable alluvial deposits that surround the hill in the south. A smaller amount of groundwater flows even more to the SE towards the mineral springs (their discharge is about 1 m^3^/s) in correspondence of the outcrop of alluvial deposits and travertines until 58–59 m a.s.l. Finally, a fraction of groundwater flows through the travertines as the piezometric contours of Fig. 1 show.

### Groundwater chemistry

**Fig. 3:**
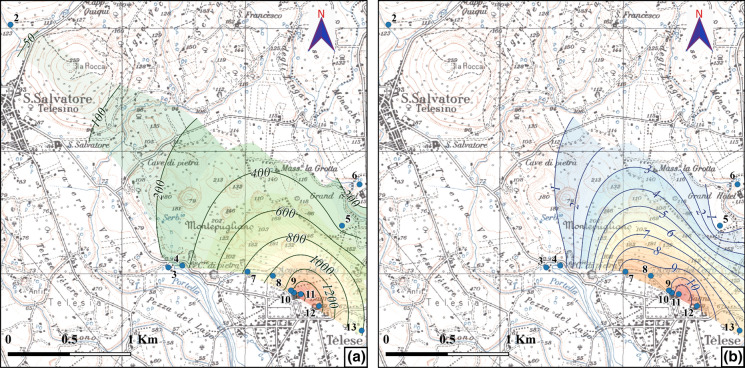
Areal distribution of CO_2_ **(a)** and H_2_S **(b)**. Concentration is reported in mg/L

Chemical analyses of groundwater evidence the highest concentrations of CO_2_ and H_2_S in the mineral waters (9–12 of Table 1) of the south-eastern sector of Montepugliano Hill. These values are present in a limited area (Fig. 3), which probably corresponds to a fault that facilitates rising up of endogenous gas.

The CO_2_ could be due to dissolution or hydrothermal metamorphism of deep limestones; in fact, the ^13^C/^12^C ratio in CO_2_ (reported as δ^13^C vs PDB) is −2.0 ‰ (Minissale et al. 2016) and falls in the range from −2.0 to + 3.0 ‰ (PDB), which typically indicates the hypothesized origin (Keith and Weber 1964; Friedman 1970). In the range from −4.0 to −7.0 ‰, CO_2_ is instead mainly originated in the mantle (Deines and Gold 1973; Javoy et al. 1984).

**Fig. 4:**
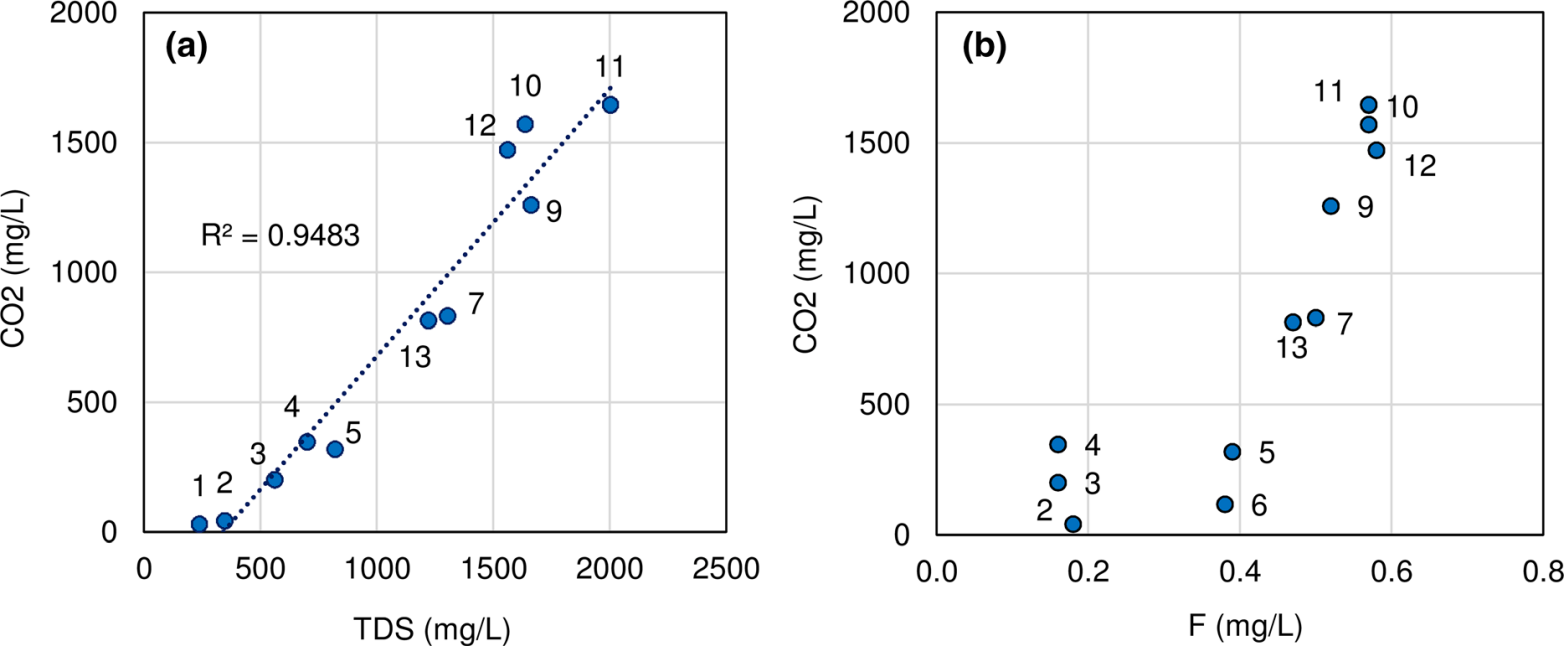
Chemical correlations of the waters sampled in November 2019 (Table 1); **a** CO_2_ vs TDS and **b** CO_2_ vs F

**Fig. 5:**
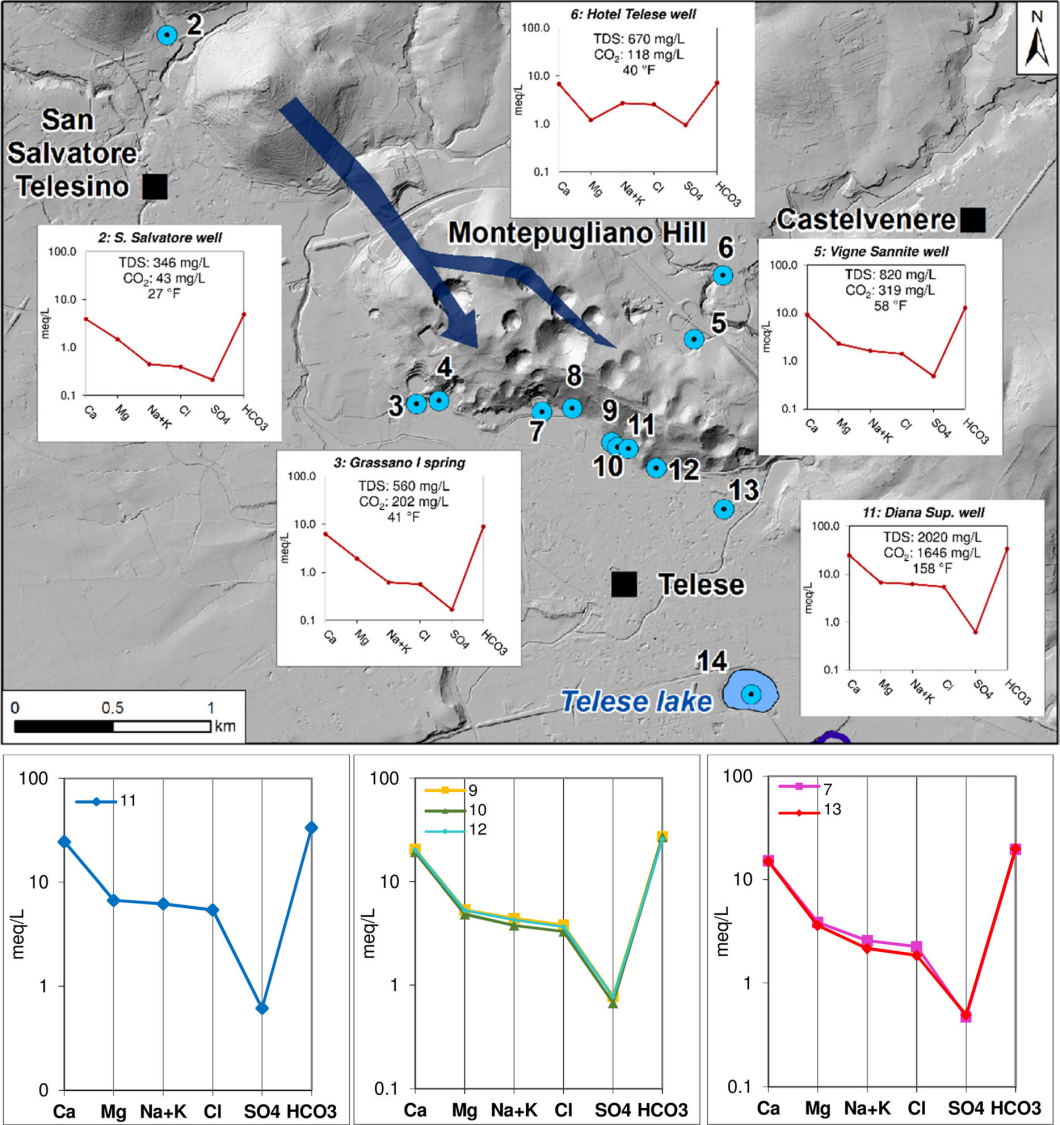
Chemistry modification (Schoeller–Berkaloff graphs) along groundwater basal flow (blue arrow) in the carbonate hills (Table 1) and below, graphs of mineral waters: (11) Diana Superiore well; (9) S. Lucia spring; (10) Diana Inferiore well; (12) Goccioloni well; (7) Centro Relax well; (13) S. Stefano well (data of November 2019)

In the SE sector of Montepugliano Hill, where mineral waters are observed, also TDS shows the highest values, because CO_2_ increases the reactivity of groundwater towards the carbonate aquifer (Figs. 4a and  5). Moreover, CO_2_ increases the amount of the minor constituents such as fluoride (Fig. 4b and Table 2) of mineral waters.

**Table 2:** Main characteristic ionic ratios (see Table 1) and some minor constituents

*n*	Date	K/Na	(Na + K)/Cl	Mg/Ca	SO4/Cl	(Ca + Mg)/(Na + K)	*B*	*F*	*Si*	*Sr*	*Li*	*Br*
							*mg/L*	*mg/L*	*mg/L*	*mg/L*	*mg/L*	*mg/L*
1	01/10/1985 (b)	0.02	0.70	0.20	0.48	15.31	–	–	–	–	–	–
2	10/11/2019 (a)	0.14	1.13	0.37	0.54	12.24	< 0.1	0.18	–	–	0.01	0.11
3	01/02/1976 (b)	0.17	0.45	0.24	0.15	25.65	–	–	–	–	–	–
	26/11/2019 (a)	0.12	1.09	0.30	0.30	13.38	< 0.5	0.16	8.20	0.23	0.05	0.26
	20/01/2020 (a)	0.11	1.22	0.30	0.43	13.28	< 0.5	< 0.1	9.45	0.22	0.06	0.04
	20/02/2020 (a)	0.11	1.23	0.30	0.33	13.47	< 0.5	0.12	8.16	–	0.06	0.40
4	20/06/2003 (c)	0.10	1.31	0.29	0.31	14.64	–	–	–	–	–	–
	02/12/2003 (c)	0.09	1.32	0.36	0.30	11.80	–	–	–	–	–	–
	25/05/2005 (c)	0.12	0.84	0.28	0.17	12.06	–	–	–	–	–	–
	20/09/2005 (c)	0.10	1.09	0.30	0.34	12.51	–	–	–	–	–	–
	05/06/2007 (c)	0.11	1.12	0.30	0.26	13.51	–	–	–	–	–	–
	10/10/2007 (c)	0.09	1.27	0.32	0.28	13.70	–	–	–	–	–	–
	04/06/2009 (c)	0.12	1.54	0.30	0.26	13.00	–	–	–	–	–	–
	03/12/2009 (c)	0.09	1.17	0.29	0.28	14.48	–	–	–	–	–	–
	16/12/2010 (c)	0.10	0.97	0.30	0.25	14.54	–	–	–	–	–	–
	15/05/2012 (c)	0.13	1.54	0.30	0.33	13.27	–	–	–	–	–	–
	25/10/2012 (c)	0.11	1.05	0.29	0.30	14.05	–	–	–	–	–	–
	26/11/2019 (a)	0.10	1.08	0.26	0.25	13.14	0.50	0.16	7.90	0.23	0.05	0.26
	20/01/2020 (a)	0.09	1.17	0.25	0.32	13.06	0.60	0.16	7.15	0.27	0.10	0.09
	20/02/2020 (a)	0.09	1.16	0.25	0.26	13.08	0.50	0.12	6.82	–	0.09	0.52
5	26/11/2019 (a)	0.09	1.15	0.24	0.34	7.20	0.70	0.39	13.90	0.41	0.11	0.48
	22/01/2020 (a)	0.10	1.21	0.25	0.40	8.40	0.80	0.33	12.90	0.36	0.14	0.09
	20/02/2020 (a)	0.10	1.18	0.25	0.35	8.63	0.60	0.31	12.00	–	0.12	0.61
6	26/11/2019 (a)	0.14	1.06	0.17	0.37	2.99	< 0.5	0.38	39.00	0.48	0.01	0.27
	22/02/2020 (a)	0.14	0.93	0.16	0.28	2.49	< 0.5	0.27	41.10	–	–	0.60
7	26/11/2019 (a)	0.09	1.14	0.25	0.21	7.51	2.30	0.50	10.80	0.66	0.33	1.30
	20/01/2020 (a)	0.09	1.16	0.25	0.18	7.90	1.90	0.42	8.85	0.51	0.35	1.40
	20/02/2020 (a)	0.09	1.13	0.25	0.17	8.00	1.70	0.47	9.90	–	0.31	1.20
8	01/01/1986 (b)	0.13	0.50	0.38	0.35	23.82	–	–	–	–	–	–
	22/01/2020 (a)	0.10	1.20	0.25	0.47	10.07	< 0.5	0.33	9.92	0.38	0.17	–
	22/02/2020 (a)	0.10	1.20	0.25	0.42	10.18	0.90	0.27	9.82	–	0.15	0.83
9	19/11/1998 (d)	0.11	1.23	0.27	0.07	5.65	–	–	–	–	–	–
	26/11/2019 (a)	0.09	1.16	0.26	0.20	5.93	3.90	0.52	14.40	1.04	0.60	2.11
	20/01/2020 (a)	0.09	1.14	0.26	0.16	5.76	4.90	0.63	14.20	0.95	0.98	2.60
	20/02/2020 (a)	0.09	1.14	0.26	0.12	5.64	3.50	0.54	14.10	–	0.63	2.32
10	26/11/2019 (a)	0.09	1.16	0.26	0.21	6.07	3.90	0.57	14.10	0.96	0.59	2.04
	20/01/2020 (a)	0.09	1.15	0.27	0.06	5.06	3.40	0.71	16.60	0.89	0.69	3.40
	20/02/2020 (a)	0.09	1.16	0.26	0.15	5.88	3.30	0.55	13.80	–	0.59	2.16
11	26/11/2019 (a)	0.09	1.14	0.27	0.11	5.10	5.00	0.57	15.30	1.77	0.83	3.11
	20/01/2020 (a)	0.09	1.16	0.26	0.04	5.89	3.00	0.61	14.20	0.79	0.60	2.49
	20/02/2020 (a)	0.09	1.13	0.27	0.05	5.11	4.40	0.61	15.60	–	0.82	2.98
12	26/11/2019 (a)	0.09	1.15	0.25	0.20	6.48	3.00	0.58	12.80	0.95	0.51	1.84
	20/01/2020 (a)	0.10	1.19	0.25	0.18	6.41	3.40	0.64	12.90	1.03	0.68	2.19
	22/02/2020 (a)	0.09	1.18	0.25	0.16	6.34	1.90	0.54	12.90	–	0.55	1.96
13	01/10/1983 (b)	0.10	1.29	0.24	0.12	4.21	–	–	–	–	–	–
	26/11/2019 (a)	0.10	1.16	0.24	0.27	8.74	1.70	0.47	10.90	0.60	0.26	1.01
	20/01/2020 (a)	0.10	1.17	0.24	0.18	8.36	1.70	0.47	10.30	0.64	0.34	1.46
	20/02/2020 (a)	0.09	1.18	0.24	0.16	8.21	2.00	0.40	9.91	–	0.55	1.96
14	26/11/2019 (a)	0.16	1.26	0.80	0.23	3.89	0.53	0.13	–	0.21	0.08	0.31
	22/01/2020 (a)	0.16	1.30	0.69	0.32	4.35	0.50	0.10	8.23	0.16	0.08	0.09
	20/02/2020 (a)	0.16	1.25	0.68	0.23	4.40	0.50	0.10	7.86	–	0.08	0.37

1 (Well 1); 2 (San Salvatore well); 3 (Grassano I Spring); 4 (Grassano II Spring); 5 (Vigne Sannite Well); 6 (G. Hotel Telese Well); 7 (Centro Relax Well); 8 (Cerro Spring); 9 (S. Lucia Spring); 10 (Diana inf. Well); 11 (Diana sup. Well); 12 (Goccioloni Well); 13 (S. Stefano Well); 14 (Telese Lake)

The highest TDS value within mineral waters was found in the Diana Superiore well (*n.* 11). Moving from this well towards NW and SE directions, the TDS of mineral waters gradually decreases and a progressive variation in the chemical profile is observed (Fig. 5).

Moving away from the mineral waters towards NW (Fig. 5), groundwater is progressively less mineralized and the hydrochemical graphs tend to assume the typical shape of waters in contact with carbonate rocks (see graph of well *n.* 2 in Fig. 5).

Well *n. *6 (Fig. 5) has a completely different chemical profile, because the drilling (unlike the others) did not find the carbonate aquifer but only pyroclastic deposits and flysch; therefore, its chemistry has not been taken into consideration in the various elaborations.

Taking into account the hydrochemistry, concentration gradients have been observed between the two end terms represented by the mineral waters of the Diana Superiore well (*n.* 11 in Fig. 5) and those of the wells *n.* 2 (Fig. 5). The other waters have intermediate chemical composition. Several studies have used chemical compounds in simple mixing models (also known as end-member mixing analysis, EMMA) to quantify the amounts of the different components of a binary mixing (Christophersen and Hooper 1992). Generally, greater is the difference between concentrations, greater is the sensitivity and lower is the uncertainty of EMMA approach (Genereux 1998; Phillips and Gregg 2000). In addition, end-member concentrations must be relatively constant in both space and time over the period of interest. Applying the end-member mixing analysis to the water sampled in November 2019, considering as end-members the wells *n.* 2 and *n.* 11 and using [Cl^−^] as conservative tracer, the following percentage (in parenthesis) of freshwater coming from the well *n.* 2 has been calculated in the different wells: *n.* 3 (97%), *n.* 4 (93%), *n.* 7 (63%), *n.* 9 (32%), *n.* 10 (35%), *n.* 12 (42%), *n.* 13 (71%), *n.* 14 (92%).

**Fig. 6:**
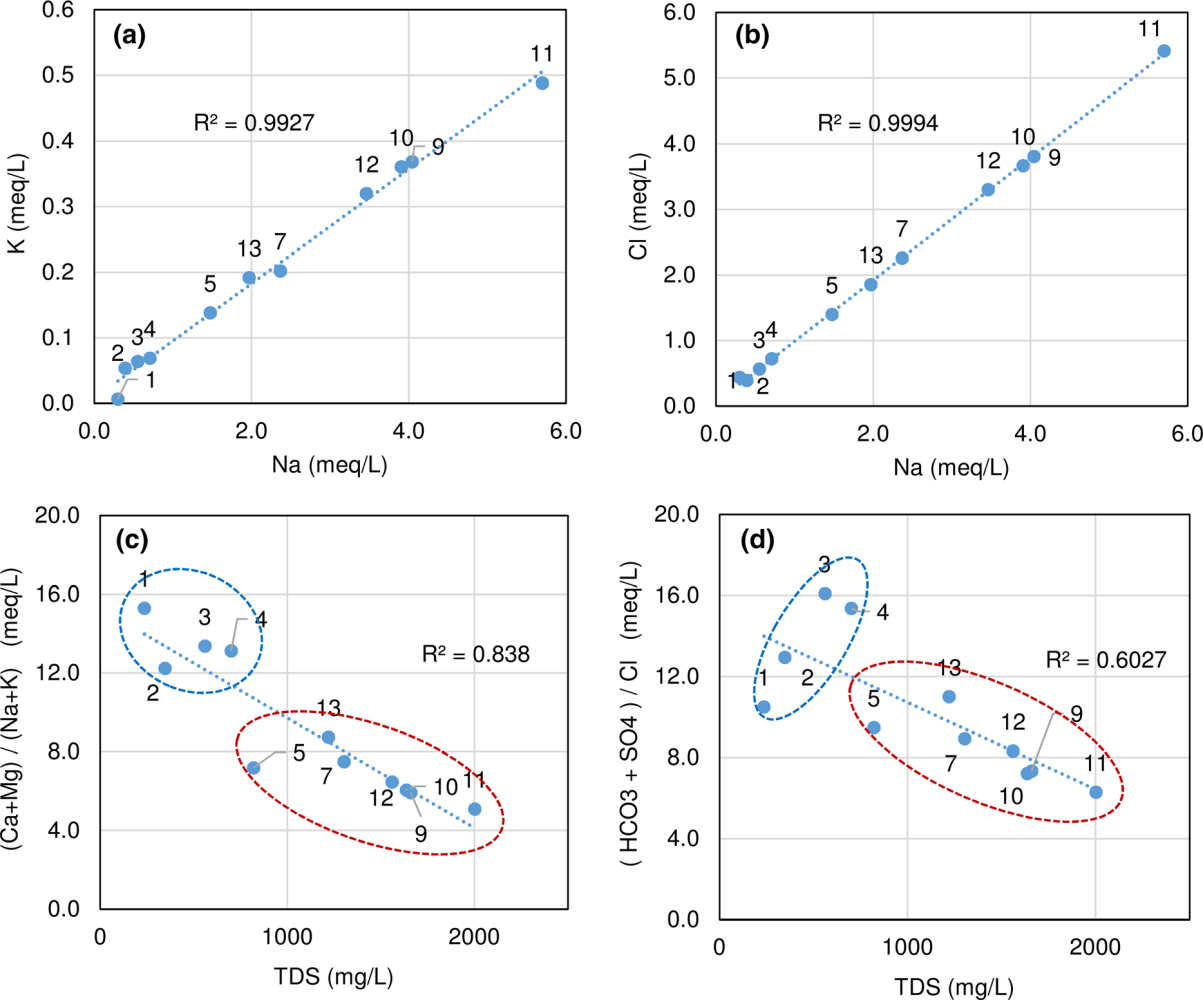
Hydrochemical graphs (data of November 2019, Table 1)

In all the waters, Na is very well correlated with K and Cl (Fig. 6); however, moving from the waters with low TDS values (from *n.* 1 to 4) to the mineral waters, the alkaline metals increase proportionally more than Ca and Mg; the same happens for Cl with respect to other anions (Table 2). In fact, the ionic ratios r[(Ca + Mg)/(Na + K)] and r[(HCO_3_ + SO_4_)/Cl] are significantly lower in mineral waters and divide these waters from all the others (Fig. 6). This feature has also been confirmed in the samplings of January and February 2020.

**Fig. 7:**
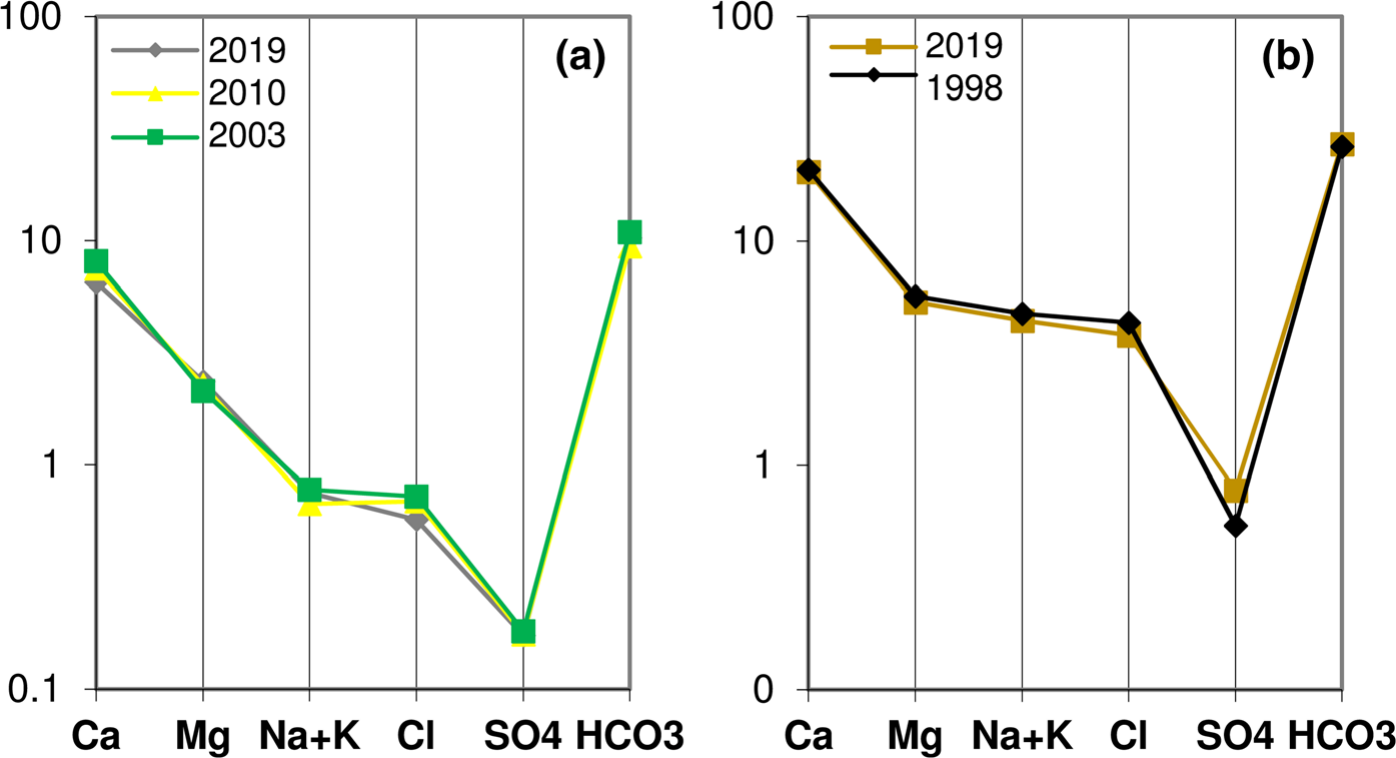
Schoeller–Berkaloff graphs (data in meq/L, Table 1) of the spring n. 4 (Grassano II), analyses of December 2003, 2010 and 2019 **(a)**, and of the mineral spring n. 9 (S. Lucia), analyses of November 1998 and 2019 **(b)**

Over time, the chemistry of the Grassano springs (*n.* 3 and 4) appears very constant when comparing analyses performed in the same periods of different years (Fig. 7a). The same constant trend is found for mineral waters as shown in Fig. 7b.

**Fig. 8:**
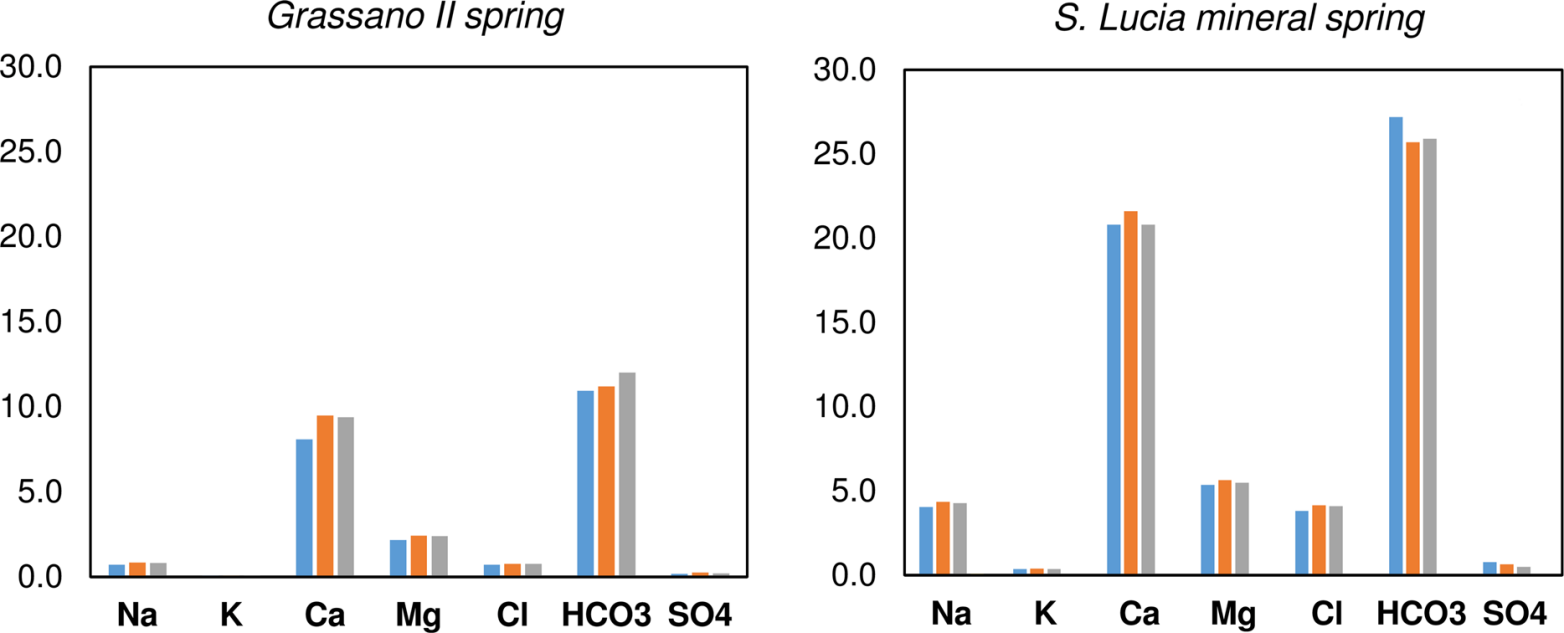
Chemical data (major ions in meq/L) of November 2019 (blue), January (orange) and February 2020 (grey)

The monthly physico-chemical analyses performed for this study (from November 2019 to February 2020) did not provide useful indications on chemical variations over time. In the area of interest, November generally marks the beginning of a rainy period that extends over several months. Nevertheless, for the considered period, only November and December were rainy months. Therefore, the concentrations of the various ions have remained almost constant or present slight variations (Table 1; Fig. 8).

### Isotopic data

Water stable isotopes may infer information about the origin and the recharge processes of groundwater (Clark and Fritz 1997), as well as mixing issues (Petrella and Celico 2013). Water isotope data in this work refer to the sampling campaign of November 2019, when 12 samples of springs, wells and lake water have been collected. The δ^18^O and the δD values range from −8.6 to −0.6 ‰ and from −49.6 to −10.3 ‰, respectively. The Telese lake (*n.* 14) is characterized by the most enriched values due to evaporation of the lake surface, while the Grassano springs (*n.* 3 and 4) are characterized by the most depleted values (Table 1).

**Fig. 9:**
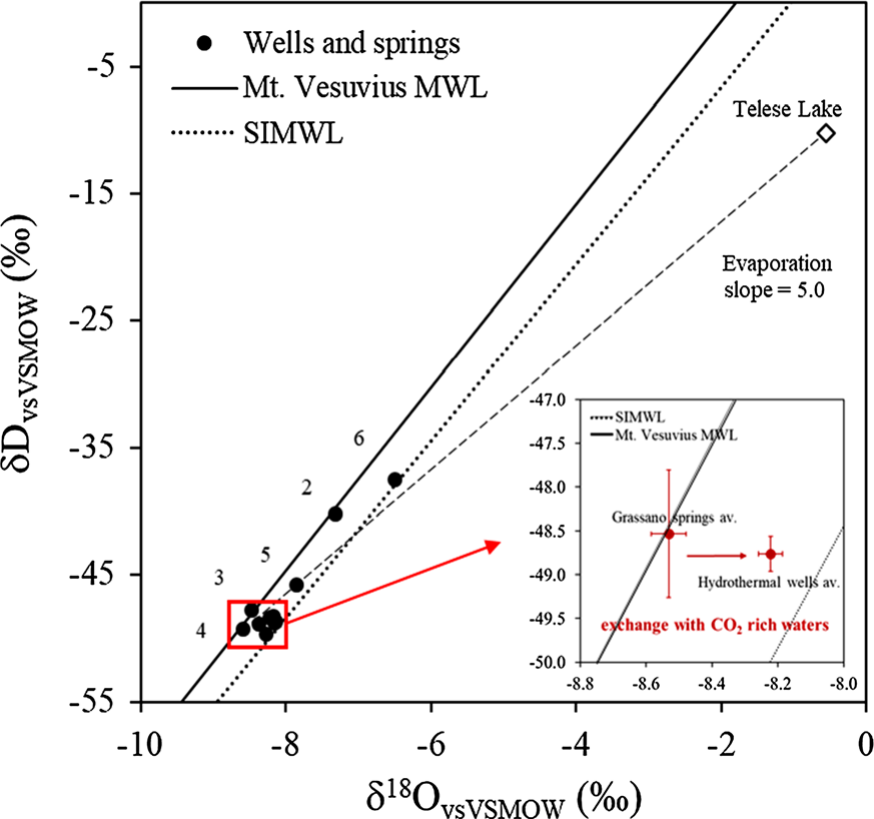
δD vs δ^18^O diagram of springs, wells and lake water. In the small box, the average values of two groups of waters (n. 3. and n. 4 Grassano springs; n. 7 to n. 12 hydrothermal waters) are represented in red

In Fig. 9, the water samples are compared with the South Italy Meteoric Water Line (MWL, Longinelli and Selmo 2003) and the Meteoric Water Line of the Vesuvius Mt. (Madonia et al. 2014), which is located about 50 km to the south from the study site. In the graph, the springs and wells (*n.* 3–4–7–9–10–11–12–13) located at Montepugliano Hill plot close to the Vesuvius MWL. Those sampling locations are characterized by more depleted values of δD and δ^18^O indicating a common recharge from rain infiltrating at a higher quote with respect to the wells *n.* 2–5–6, which have more enriched isotopic values. As already noted, well *n.* 6 does not meet the carbonate aquifer and the nearby well *n.* 5 is partly affected by this local setting. Conversely, well *n.* 2 located at the base of Acero Mt. drains the same groundwater that feeds Montepugliano area; its isotopic values can be related by the mixing between this groundwater and the waters of surface infiltration due to the rains that preceded the sampling. The group of the mineral waters (wells *n.* 7–9–10–11–12–13) has mean δ^18^O values significantly different from the average Grassano springs values (> 2 sigma): −8.5 ± 0.1‰ versus −8.22 ± 0.04‰, respectively. As reported in the box inside the plot in Fig. 9, a shift in δ^18^O values is observed. This trend is likely due to the mixing with CO_2_-rich waters (Table 1 and Fig. 3) which can cause a horizontal deviation from the MWL (D'Amore and Panichi 1987; Pauwels et al. 2007; Cartwright et al. 2000; Karolyté et al. 2017). In fact, CO_2_ can promote low temperature mineral dissolution and secondary mineral precipitation reactions, preferentially consuming ^18^O and equilibrium oxygen isotope exchange between CO_2_ and water, leaving the δD signature unchanged. Taking into account this process, the average infiltration altitude can be hypothesized the same as the Grassano springs.

Applying the empirical equation for the δ^18^O altitude gradient (*z* = −512.24* δ^18^O −2781) found by Di Luccio et al. (2018) for the Matese massif which is close to the study area (see Fig. 1), the mean recharge altitude of the waters of Montepugliano (springs and well from 7 to 13) is about 1500 m a.s.l.

The waters sampled in Telese lake show a strongly enriched isotopic signature typical of an evaporation process taking place at lake surface. The evaporation line is characterized by a slope of 5.0 if we assume that the lake is mainly fed by groundwater coming from the upgradient Grassano springs and in general from the Matese massif.

**Fig. 10:**
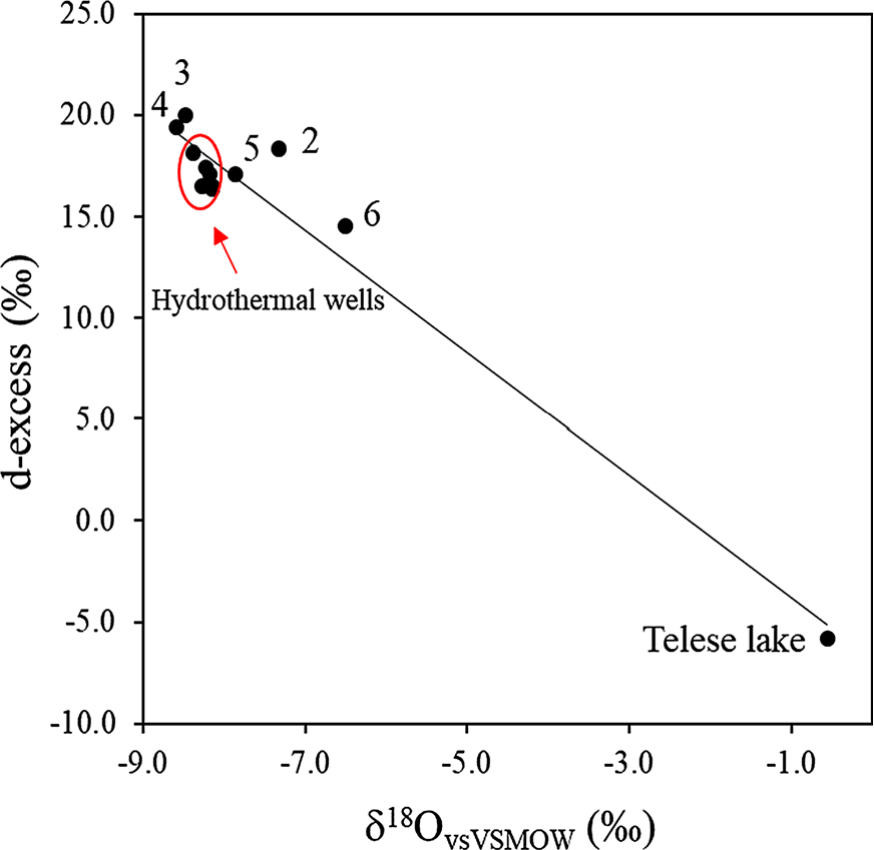
Deuterium excess versus δ^18^O of springs and wells. The group without labels refer to hydromineral waters (n. 7–9–10–11-12–13)

Finally, the deuterium excess (d_excess_ = δD–8*δ^18^O, Dansgaard 1964) is a parameter used to infer information about water vapour formation originating the precipitation (Merlivat and Jouzel 1979; Froehlich et al. 2002; Pfahl and Sodemann 2014). Also in this case (Fig. 10), the similar values of the springs and wells (from 7 to 13) indicate homogeneous recharge conditions. For this study area, Grassano springs have the highest d_excess_ values indicating a recharge from air masses formed in conditions of low relative humidity, typical of the Mediterranean basin. This hypothesis is further supported by the closeness of Grassano spring isotopic signature to the Mt. Vesuvius MWL (Fig. 9), indicating a recharge occurring from rains originating from the western Mediterranean basin.

### Microbiota characterization

In the sampled waters, 390,873 reads were considered (Table 3) by 16S rRNA sequencing after removed the low-quality, non-chimeric and non-bacterial reads.

**Table 3:** Total number of sequencing reads vs EC for the sampled waters

Well/spring	Total number of sequencing reads	Cond µS/cm
2	23,528	501
3	16,985	800
4	14,206	980
5	19,601	1173
6	8730	969
7	39,005	1862
9	23,383	2391
10	66,448	2338
11	73,858	2884
12	53,644	2256
13	51,481	1730

**Fig. 11:**
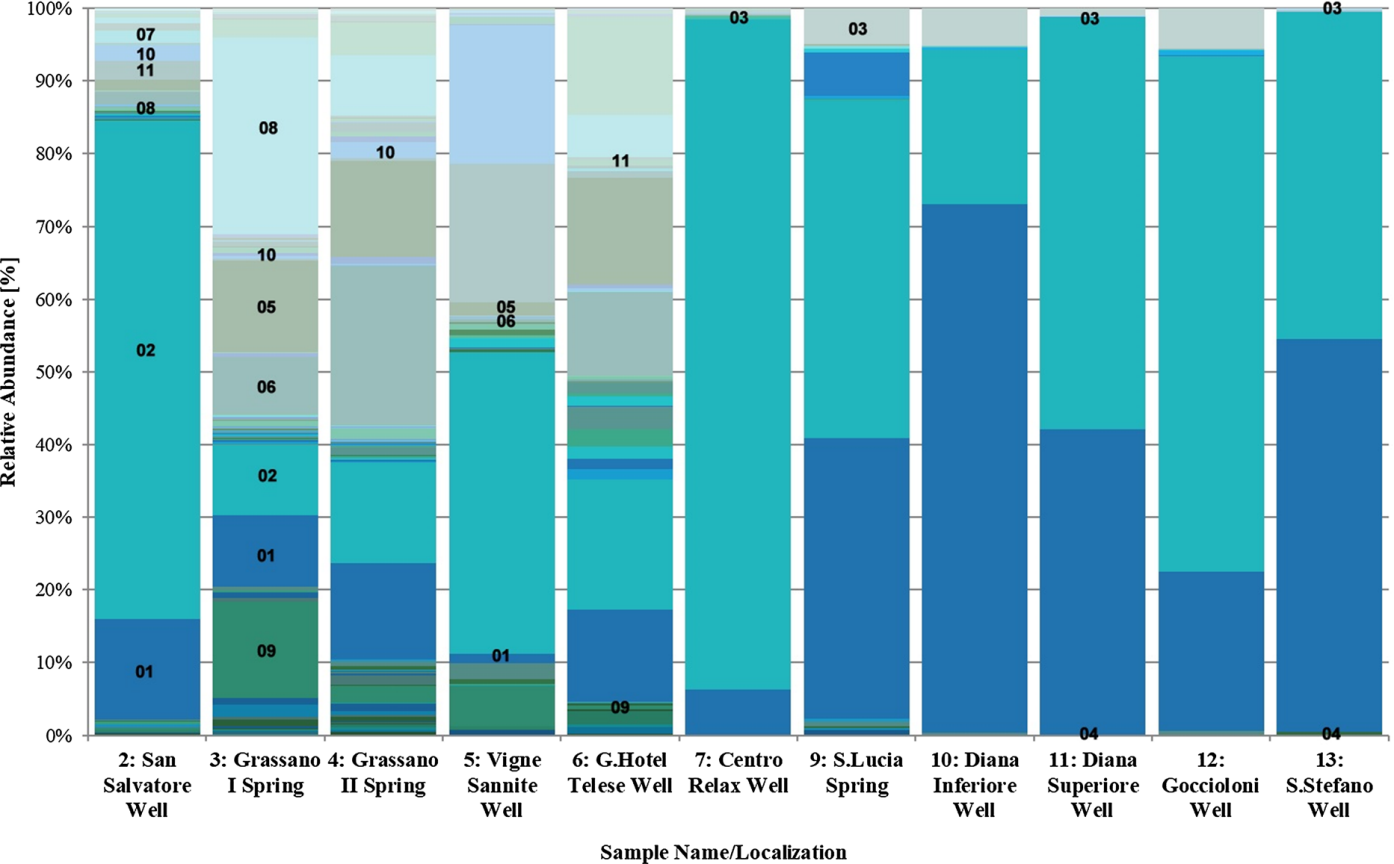
Barplot of the sampled waters at Family level according to 16 s rRNA sequencing analysis. Results are expressed as relative abundance of reads. (01) *Sulfurovaceae;* (02) *Thiovulaceae*; (03) *Halothiobacillaceae*; (04) *Caldisericaceae*; (05) *Burkholderiaceae*; (06) *Sphingomonadaceae*; (07) *Diplorickettsiaceae*; (08) *Moraxellaceae*; (09) *Flavobacteriaceae*; (10) *Hydrogenophilaceae*; (11) *Gallionellaceae*

The highest abundance, at Family level, was observed in all samples, excepting sample *n.* 5, for *Sulfurovaceae* (code 01, Fig. 11) and *Thiovulaceae* (code 02, Fig. 11). Beyond the two above-mentioned families, the composition is different in two specific groups.

The more mineralized waters (7–13) showed the higher sequences count (Table 3) but also the lower diversity in terms of microbial Families (Fig. 11). These waters have the prevalent abundance of *Sulfurovaceae* (from 6 to 73%) and *Thiovulaceae* (from 21 to 92%) families. *Halothiobacillaceae* family (code 03, Fig. 11) was detected in all samples (from 1 to 5%), excepted for sample 13, where *Caldisericaeae* family (code 04, Fig. 11) is the third most abundant family.

The second group included samples *n.* 2, 3, 4 and 6, where *Burkholderiaceae* (from 1.5% to 15%, code 05, Fig. 11) and *Sphingomonadaceae* (from 2 to 22%, code 06, Fig. 11) families were the most abundant, after *Sulfurovaceae* and *Thiovulaceae*. Wells *n.* 2 and *n.* 6 were discriminated thanks to *Pseudomonadaceae* family abundance (13% in well *n.* 6) and to the presence of *Diplorickettsiaceae* (2% in well *n.* 2, code 07, Fig. 11). Samples *n.* 3 and *n.* 4 (Grassano springs I e II) are the closest to each other: they are additionally composed by *Moraxellaceae* (8% in sample *n.* 4; 27% in sample *n.* 3, code 08, Fig. 11) and *Flavobacteriaceae* (2.5% in sample *n.* 4; 13% in sample *n.* 3, code 09, Fig. 11); moreover, *Hydrogenophillaceae* family (code 10, Fig. 11) is exclusively present in sample *n.* 4 (2.2%).

**Fig. 12:**
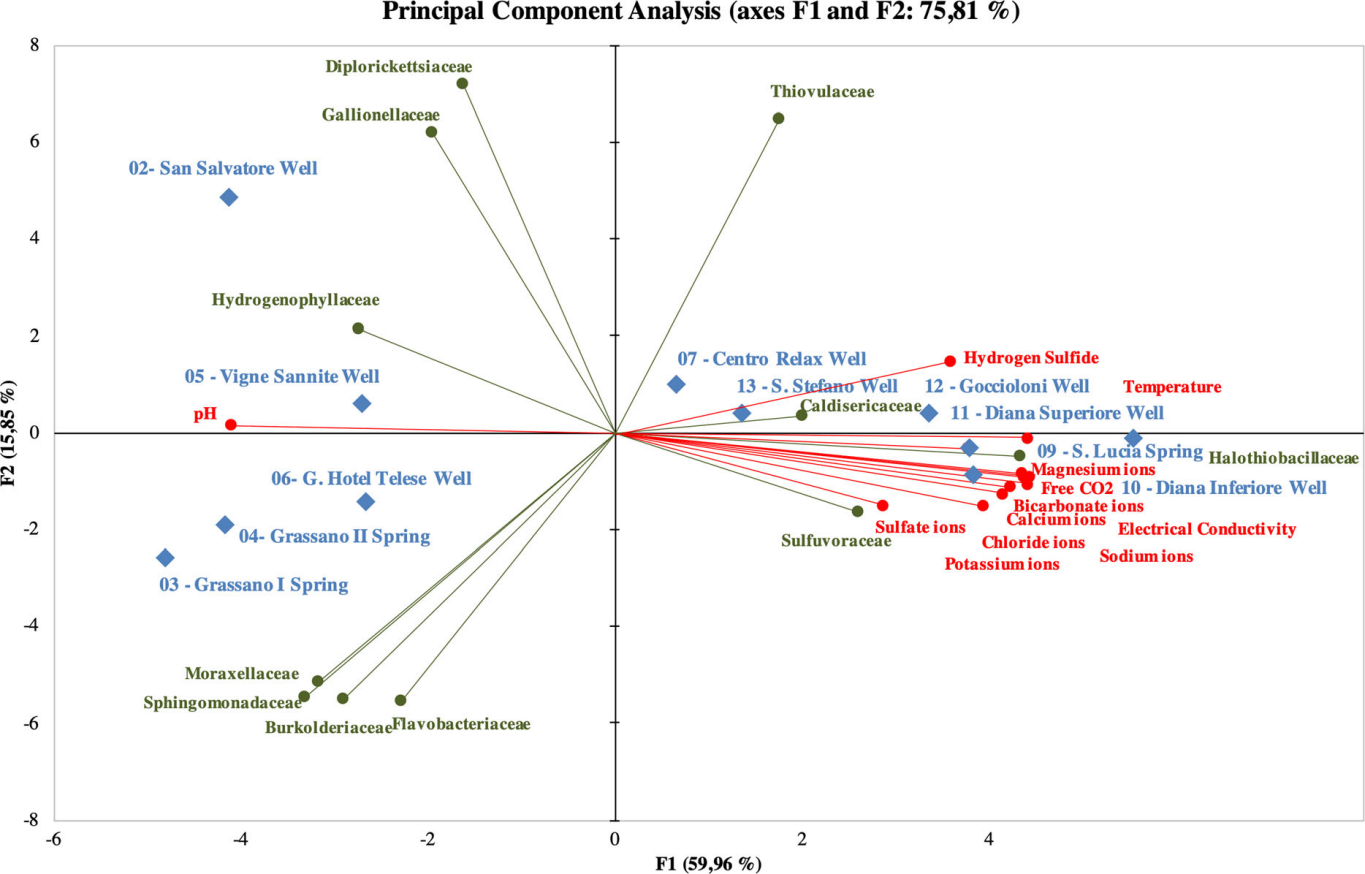
Principal components analysis (PCA) with the MiSeq Illumina fingerprints, associated with chemical data. F1 and F2 are shown on x and y axes, respectively

Finally, well *n.* 5 is very different from the all other waters: indeed, it is characterized by the *Gallionellaceae* (19%, code 11, Fig. 11), *Hydrogenophillaceae* (19%), *Flavobacteriaceae* (5.5%) beyond *Thiovulaceae* (41.5%), the only family in common with the other waters. This is in very good accordance with the differences that these waters show under the chemical and isotopic profiles. A multivariate analysis of MiSeq Illumina fingerprints associated with metadata was carried out. Figure 12 shows principal component analysis (PCA) of the relative abundance of each bacterial family (green) across the thirteen sampling sites (blue). The lines represent the correlation coefficient between the principal component scores and each environmental parameter (red). It showed that the first two principal components (F1 and F2) accounted for more than 75% of the variation in taxonomic composition among samples, and samples clustered into two major groups: waters with low TDS (2, 3, 4, 5, 6) vs. mineral waters (7, 9, 10, 11, 12, 13). The first group is more abundant in family types and is dominated by *Diplorickettsiaceae, Gallionellaceae, Hydrogenophillaceae, Moraxellaceae, Sphingomonadaceae, Burkholderiaceae* and *Flavobacteriaceae*. The second group is mainly dominated by three families, i.e. *Caldisericaeae, Halothiobacillaceae* and *Sulfurovaceae.* Most environmental parameters (except pH) show positive correlations with the F1 scores and are densely plotted on the right-hand side of the plot. PCA supports the hypothesis of the existence of distinct bacterial communities and compositions, in terms of microbiota and chemical parameters.

## Discussion

The isotopic data clearly show that Grassano springs and the various mineral waters are fed by the same catchment area, which corresponds to the south-eastern sector of the Matese massif. In particular, the isotopic data evidence a common recharge by rains originating from the western Mediterranean basin infiltrating at an average altitude of 1500 m a.s.l. The wells *n.* 2–5–6 show a distinct isotopic signature due to various factors (different recharge area, mixing with shallow groundwater, etc.

Groundwater flows from Matese to Montepugliano Hill through the carbonate bedrock below Titerno River plain (well *n.* 1 in Fig. 2) and at the foothill of Acero Mt, where (see well *n.* 2, Fig. 1 and Fig. 5) the chemical characteristics of these waters are typical of waters in contact with carbonate rocks (Corniello 1996, Fig. 5). Well *n.* 2, only 3 km far from the most mineralized well (Diana Superiore *n.* 11—TDS: 2020 mg/L; Fig. 5), has in fact TDS = 346 mg/L. At Grassano I spring (*n.* 3; 900 m from the Diana Sup. well), the TDS is 560 mg/L and 820 mg/L in the well *n.* 5 (about 600 m far from the Diana Sup. well). The mineral springs/wells are also distributed at the basis of the Montepugliano Hill (Figs. 3 and  5) in a narrow area, which probably corresponds to a fault line connected to the regional faults of the Calore River valley.

Groundwater flow that feeds all the waters mentioned above is unique and it is directed from NW to SE (Fig. 5); therefore, mineralization occurs only in the final part of the groundwater flow path, where uprising of deep gases (CO_2_ and H_2_S, Figs. 4 and 5, Table 1) occurs along the faults of Montepugliano Hill. A frequently observed mineralization pattern (Goldscheider et al. 2010; Corniello 1996; Corniello et al. 2013, 2018) therefore would be confirmed also for the Telese mineral waters. All the waters of the Montepugliano area, hence, result from the mixing (in different percentages) of the end terms represented by the waters of the wells *n.* 2 and the well *n.* 11, as reported by the EMMA analysis using chloride as a conservative tracer of the mixing.

The rising up of deep gas within carbonate rocks should not change the chemical profile of groundwater but only increase their concentration (Corniello 1996). As regards the sulphurous waters of Telese, an increase also of alkaline ions and Cl^−^ is observed (Table 2); this means that along the faults with gas also long-circulating deep waters rise, in which Cl^−^, however, becomes important, even without evaporites and/or marine waters contribution (Chebotarev 1955; Minissale et al. 2016).

As previously indicated (Fig. 1), numerous sinkholes are present on the Montepugliano Hill and their areal concentration is certainly due to the local CO_2_-rich mineral waters, which promote rock dissolution. However, this gas is heavier than air and the bottom of the sinkholes is, on average, about 60 m (data by Fiorillo et al. 2019) higher than mineral springs. To explain the influence of these waters on the karst; therefore, it is necessary to consider one or more variations of the *karst base level*. One of these variations may have been linked to the deposition of thick tuff layer (39,000 y b.p.; De Vivo et al. 2001) at the base of Montepugliano hill and of the other nearby reliefs. This deposition blocked the natural outflow of the groundwater and resulted in a gradual raising of the water table within the reliefs and a higher *karst base level* at the top of the tuff. The gas-rich waters have therefore affected the highest altitude sectors of Montepugliano Hill and determined the development of the karst phenomenon currently observed. Subsequent erosion, due to the copious spring flow, would have led the springs up to the current altitude. In this regard, the absence of tuff only near the springs is a significant clue supporting the latter hypothesis; very thick tuff layer instead still outcrops on the other slopes of Montepugliano Hill (Fig. 1).

**Fig. 13:**
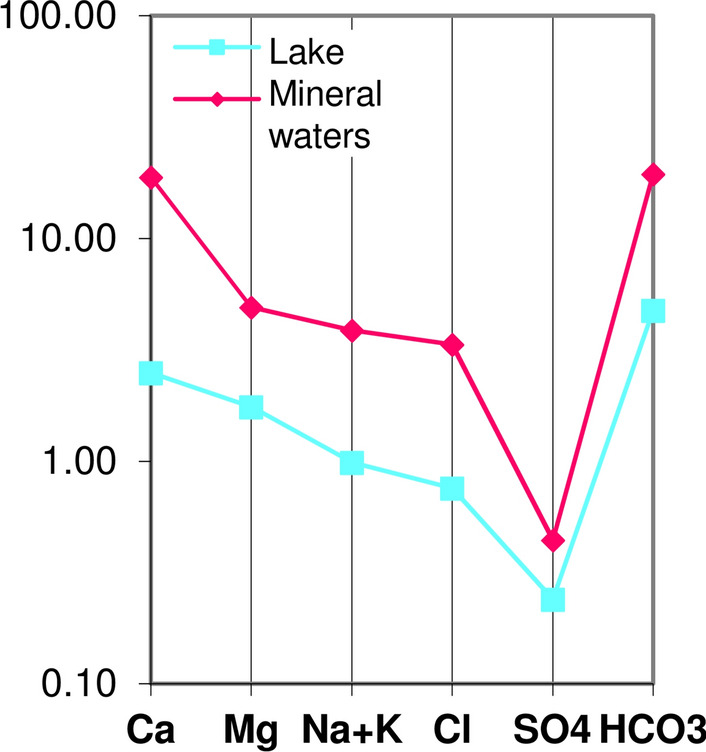
Schoeller–Berkaloff graph (meq/L); lake (down) and the mean values of the mineral waters (January 2020—Table 1)

Also the Telese lake is hosted in a wide sinkhole in the travertines (Fig. 1) of the plain south of the hill. The lake is fed by underground flow from the Montepugliano Hill to the travertines as the piezometric curves of Fig. 1 and hydrochemistry (EMMA analysis) indicate. The waters of the lake have a chemical profile very different from that of the mineral waters (Fig. 13), and in particular, they have much lower TDS (Table 1) and higher values of the rMg/rCa ratio (Table 2). These differences, probably, are due to *(a)* CaCO_3_ removal affecting the mineral waters on the way from the hill to the lake whose waters show positive value (November 2019) of the *Langelier index* and *(b)* underground contributions from the Grassano springs.

In Montepugliano Hill, there is not only a change of the groundwater chemistry but also of their microbiome, and also in this case, the variations occur in the final part of the groundwater flow. In fact, waters with low microbial abundance and higher diversity (well *n.* 2, Grassano springs *n.* 3 and *n.* 4) become characterized by high microbial abundance and lower diversity (clear prevalence of *Sulfurovaceae* and *Thiovulaceae*) in the mineral zone.

On the other hand, groundwater velocity makes possible a progressive adaptation of the microbiota to the new environmental conditions. For example, the distance (about 600 m) from Grassano springs to the nearest mineral well (relax centre *n.* 7) would be covered in around one month: a time consistent with those indicated by Valeriani et al. (2020) studying the microbiota variations over time due to an earthquake at Ischia island.

At Telese, the influence of temperature on microbiota variation is probably negligible since it varies from 12.3 °C in the well *n.* 2 to 21 °C in the most mineralized well (Diana Sup. *n.* 11). Contrary to water samples taken from 1 to 4, mineral waters show significant increases of TDS and of some minor constituents such as Sr, I, B, Li and Br (Table 2); most of all, microbiota is influenced by the hydrogen sulphide. In fact, H_2_S is measured only in mineral waters (Table 1) and its cellular toxicity is known and allows the survival of only few species of micro-organisms (Valeriani et al. 2018). Free carbon dioxide may additionally influence microbial composition and variety: CO_2_ in mineral waters varies from 815 to 1646 mg/L, while much lower concentrations are present in the other sampled waters (from 42 to 348 mg/L).

Finally, as reported by Pedron et al. (2019) for the mineral waters of Comano (Italy), also in the microbiota of Telese mineral waters, a difference is observed comparing the waters sampled in wells with those from springs (S. Lucia *n.* 9).

## Concluding remarks

The multidisciplinary study conducted for the springs and sulphurous waters of Telese has brought very useful results and can therefore constitute a cognitive approach to be replicated also in other hydrogeological settings.

The employment of isotopic data confirmed that all the mineral and non-mineral waters at the base of Montepugliano Hill are fed by the same hydrogeological basin corresponding to a wide south-eastern sector of the Matese massif; this evidence contributes to eliminating the residual uncertainties in this regard.

Further insights are provided on the process leading to the formation of sulphurous and CO_2_-rich waters used in *Telese Spa*. In fact, the groundwater coming from Matese becomes *mineral water* in the final part of the underground flow where it mixes with the rise up of deep gases (CO_2_ and H_2_S) along the faults at the southern base of Montepugliano Hill. Moving away from these faults, the mineralization decreases; in fact, groundwater sampled in well *n.* 2 (upstream and about 3 km far from the mineral area) is calcium bicarbonate type, cold and with low TDS.

The multidisciplinary study of this area permitted to define the mineralization scheme, which becomes very important due to the high volumes of water resources available. In this case, it is possible to exploit both mineral waters in Spa and the non-mineralized waters far from the faults that determine the gas uprising. In this setting, the correct quantitative management of the resources is strongly related to the knowledge of the hydrogeological basin.

Finally, the application of the microbiota analysis provided very interesting results summarized in the following points:

- in the comparison between non-mineralized waters and mineral waters, the latter show higher microbial abundance and lower diversity (in this case prevalence of *Sulfurovaceae* and *Thiovulaceae* families) which would seem due to TDS and to the presence of H_2_S (and *p.p.* of CO_2_). A further step in understanding the influences of chemistry on the microbial community would be the comparison with other microbiota data present in sulphurous waters having both similar and different physico-chemical characteristics. Currently in Campania, but also in Italy, metagenomic analyses are still few despite the presence of several sulphurous waters;
- the detection of microbial community in mineral waters may be of interest for public health purposes (e.g. for the properties and use of muds; Paduano et al. 2018);
- a specific barcode defined for the Telese sulphurous waters could support an efficient qualitative monitoring of the mineral resources and provide a basis for comparison with other mineral waters.

## Data Availability

Not applicable.
